# Menopause causes metabolic and cognitive impairments in a chronic cerebral hypoperfusion model of vascular contributions to cognitive impairment and dementia

**DOI:** 10.1186/s13293-023-00518-7

**Published:** 2023-05-23

**Authors:** Olivia J. Gannon, Janvie S. Naik, David Riccio, Febronia M. Mansour, Charly Abi-Ghanem, Abigail E. Salinero, Richard D. Kelly, Heddwen L. Brooks, Kristen L. Zuloaga

**Affiliations:** 1 Department of Neuroscience and Experimental Therapeutics, Albany Medical College, 47 New Scotland Avenue, MC-136, Albany, NY 12208 USA; 2 Department of Physiology, University of Arizona College of Medicine, Tucson, AZ 85724 USA

**Keywords:** Neuroscience, Vascular, Dementia, Menopause, Cognitive impairment, Hypoperfusion, Estrogen, Metabolic, Myelin, Neuroinflammation

## Abstract

**Background:**

The vast majority of women with dementia are post-menopausal. Despite clinical relevance, menopause is underrepresented in rodent models of dementia. Before menopause, women are less likely than men to experience strokes, obesity, and diabetes—known risk factors for vascular contributions to cognitive impairment and dementia (VCID). During menopause, ovarian estrogen production stops and the risk of developing these dementia risk factors spikes. Here, we aimed to determine if menopause worsens cognitive impairment in VCID. We hypothesized that menopause would cause metabolic dysfunction and increase cognitive impairment in a mouse model of VCID.

**Methods:**

We performed a unilateral common carotid artery occlusion surgery to produce chronic cerebral hypoperfusion and model VCID in mice. We used 4-vinylcyclohexene diepoxide to induce accelerated ovarian failure and model menopause. We evaluated cognitive impairment using behavioral tests including novel object recognition, Barnes maze, and nest building. To assess metabolic changes, we measured weight, adiposity, and glucose tolerance. We explored multiple aspects of brain pathology including cerebral hypoperfusion and white matter changes (commonly observed in VCID) as well as changes to estrogen receptor expression (which may mediate altered sensitivity to VCID pathology post-menopause).

**Results:**

Menopause increased weight gain, glucose intolerance, and visceral adiposity. VCID caused deficits in spatial memory regardless of menopausal status. Post-menopausal VCID specifically led to additional deficits in episodic-like memory and activities of daily living. Menopause did not alter resting cerebral blood flow on the cortical surface (assessed by laser speckle contrast imaging). In the white matter, menopause decreased myelin basic protein gene expression in the corpus callosum but did not lead to overt white matter damage (assessed by Luxol fast blue). Menopause did not significantly alter estrogen receptor expression (ERα, ERβ, or GPER1) in the cortex or hippocampus.

**Conclusions:**

Overall, we have found that the accelerated ovarian failure model of menopause caused metabolic impairment and cognitive deficits in a mouse model of VCID. Further studies are needed to identify the underlying mechanism. Importantly, the post-menopausal brain still expressed estrogen receptors at normal (pre-menopausal) levels. This is encouraging for any future studies attempting to reverse the effects of estrogen loss by activating brain estrogen receptors.

**Supplementary Information:**

The online version contains supplementary material available at 10.1186/s13293-023-00518-7.

## Plain Language Summary

Nearly all women with dementia are menopausal. Reduced blood flow to the brain, resulting from damaged blood vessels, can lead to vascular dementia. Vascular dementia is the second most common cause of dementia. Before menopause, women are less likely than men to experience strokes, obesity, and diabetes—known risk factors for vascular dementia. During menopause, estrogen levels drop and the risk of developing these dementia risk factors increases. The goal of this study was to determine how menopause impacts risk factors (obesity, diabetes), memory and brain pathology in vascular dementia. This study used mouse models of vascular dementia and menopause. Menopause increased weight gain and other indicators of poor metabolic health. In mice with vascular dementia, menopausal mice had worse memory than pre-menopausal mice. After menopause, the brain still expressed estrogen receptors at normal (pre-menopausal) levels. This is encouraging for any future studies attempting to reverse the effects of estrogen loss by activating brain estrogen receptors.

**Supplementary Information:**

The online version contains supplementary material available at 10.1186/s13293-023-00518-7.

## Highlights

- Here, we examined menopausal VCID (vascular contributions to cognitive impairment and dementia) using an accelerated ovarian failure menopause model and a unilateral common carotid artery occlusion model of VCID.
- Menopause increased metabolic dysfunction (weight gain, visceral and subcutaneous fat pad weights, and impairments in glucose tolerance).
- Post-menopausal VCID mice showed a wider array of cognitive deficits, compared to controls and to those with menopause or VCID alone.
- Estrogen receptors (ERα, ERβ, and GPER1) were expressed in the cortex and hippocampus of post-menopausal mice at levels similar to pre-menopausal mice.

**Supplementary Information:**

The online version contains supplementary material available at 10.1186/s13293-023-00518-7.

## Background

Vascular contributions to cognitive impairment and dementia, VCID, are the second leading cause of dementia. VCID is the result of cerebrovascular pathology and cerebral blood flow deficits which damage the brain and contribute to cognitive impairments. Men are more likely to develop VCID than women throughout most of the lifespan, but this pattern reverses in extreme old age [1, 2]. Many VCID risk factors, such as stroke, midlife obesity, hypertension, and diabetes [2–5], develop or intensify following menopause, a midlife endocrine process involving the loss of gonadally produced estradiol. Earlier age at menopause is associated with cognitive decline [6, 7] and multiple VCID risk factors [8–12], while later age at menopause is associated with better cognitive function [13]. During natural menopause, women experience a peri-menopausal stage during which there are significant hormonal fluctuations, often accompanied by vasomotor symptoms such as hot flashes. More frequent hot flashes during peri-menopause are associated with more white matter damage [14] and cognitive impairment [15]. There is evidence to support that menopause may be a key factor in cognitive aging and impairment, yet it is not clear exactly how menopause may mediate cognitive changes particularly in VCID. Further understanding regarding the pathological development of VCID in the context of menopause may highlight areas of prevention and encourage more research into hormonal changes that occur during menopause.

There is evidence that estrogens, such as 17-β estradiol, may protect women from developing VCID [1, 2]. This is due to the many beneficial actions of estradiol on cognition, cerebrovascular function, and protection against VCID risk factors [2, 16–20]. Circulating and brain estradiol levels drop post-menopause [21] which may exacerbate pathology and thus cognitive impairment associated with VCID because estrogen is likely to protect the brain in the context of VCID. It is unknown how VCID pathology and cognitive impairment would manifest post-menopause.

Others have found that in rodent models, both ovariectomy and systemic estrogen treatment impact hippocampal gene expression [22, 23], and ERα receptors in the brain [24] and in cerebral microvessels [25]. Ovariectomy is often used to model menopause in rodents; however, this model excludes aspects of human menopause, such as a peri-menopausal period and ovarian androgen production [26]. In the current study, we modeled menopause using an ovary-intact accelerated ovarian failure model of menopause by administering the ovarian toxin 4-vinylcyclohexene diepoxide [27, 28]. The ovarian toxin 4-vinylcyclohexene diepoxide induces accelerated ovarian failure by interfering with pro-survival c-kit signaling within the ovary leading to the atresia of primordial and primary follicles [29–31]. This well characterized model leads to permanent acyclicity in rodents with few side effects [32, 33]. Recent elegant work by Blackwell et al., using this accelerated ovarian failure model of menopause, showed that menopause impairs cerebrovascular function [34]. The effects of menopause in the context of VCID or underlying vascular pathology had yet to be examined. Our prior work has found that the unilateral common carotid artery occlusion model of VCID causes long-term chronic cerebral hypoperfusion and cognitive deficits [35, 36]. Whether menopause would exacerbate cognitive deficits in VCID had not yet been tested. Thus, this is the first study to examine the effects of menopause on VCID using the more clinically relevant accelerated ovarian failure model of menopause. In the current study, we found that menopause induced metabolic impairment and caused a wider array of cognitive deficits than VCID alone.

## Methods

### Animals and experimental design

**Fig. 1:**
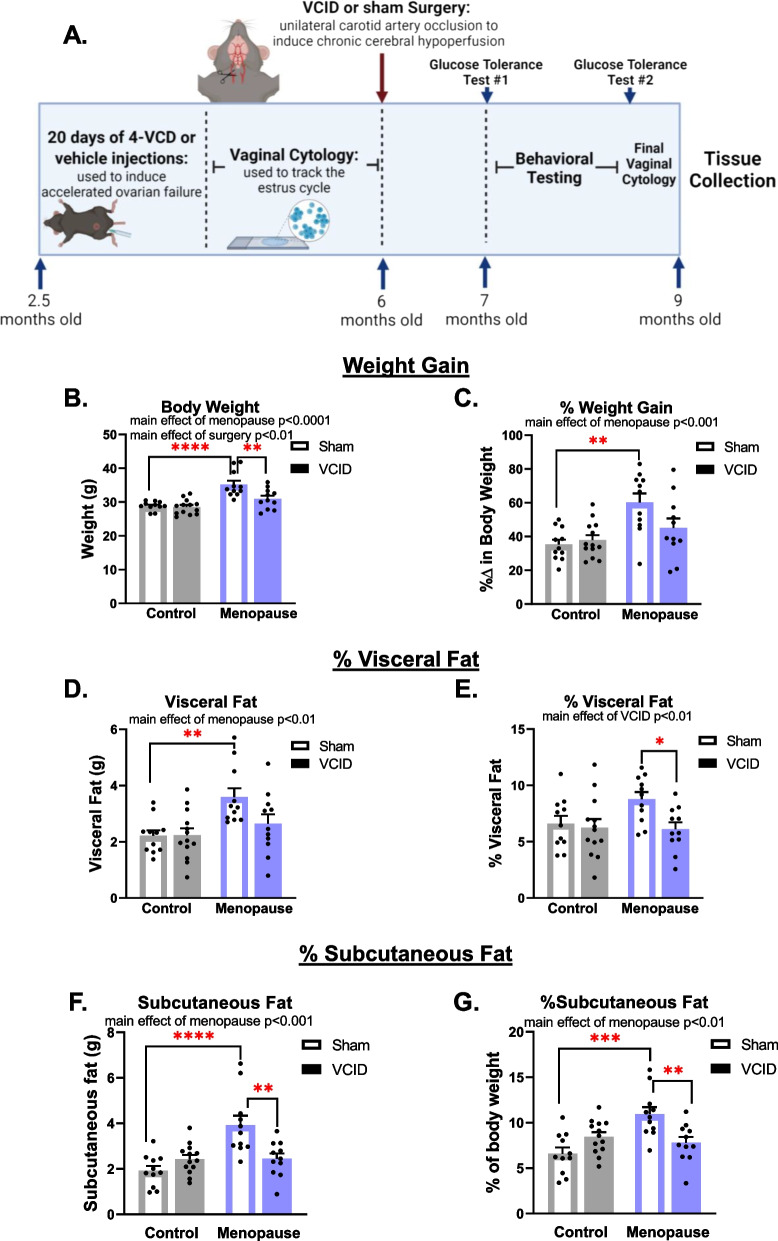
Experimental timeline and menopause-induced weight gain. **A** Experimental timeline made using Biorender.com. Body weight was taken at the end of the study (**B**) and was normalized to body weight at the beginning of the study (**C**). Visceral adiposity was determined by isolating and weighing the visceral fat pads (**D**) and normalizing to body weight (**E**). Subcutaneous adiposity was determined by isolating and weighing the subcutaneous fat pads (**F**) and normalizing (**G**) to body weight. Data are presented as mean + SEM, *p < 0.05, **p < 0.01, ***p < 0.001, ****p < 0.0001, 2-way ANOVA with Tukey’s multiple comparison test, (*n* = 10–13/group)

C57BL/6J female mice were purchased from Jackson Laboratories (Bar Harbor, ME) at 11 weeks of age and housed onsite until the start of the study. Experiments were approved by the Institutional Animal Care and Use Committee at Albany Medical College (Albany, NY, USA) and conducted in accordance with the National Institutes of Health Guidelines for the Care and Use of Laboratory Animals. Mice were fed a standard chow diet (Purina Lab Diet 5P76) and had food and water available ad libitum. They were housed in Allentown cages at a density of 2–5 mice and the facility temperature and humidity were set at 72 °F, 30–70% humidity, with a 12-h light/dark cycle (7 a.m. on/7 p.m. off). Mice were provided with environmental enrichment (Nestlets and Shepherd Shacks) and were group housed except during the nest building test. A timeline of the experiment is shown in Fig. 1A. At ~ 2.5–3 months of age, mice were given 20 days of either 4-vinylcyclohexene diepoxide or vehicle (sesame oil) and then vaginal cytology was used to track their cyclicity. Mice took on average 89.4 days to become acyclic if given 4-vinylcyclohexene diepoxide. Then at 6 months old, mice underwent a sham surgery (sham group) or a unilateral common carotid artery occlusion surgery (VCID group). Approximately 1 month later, mice underwent a glucose tolerance test (GTT), a 2-week rest period, behavioral testing, a final GTT, 10 days of vaginal cytology to confirm acyclicity, blood flow imaging, euthanasia, and tissue collection (including brains, fat, and reproductive organs). A total of 55 mice were used in this study. Experiments were conducted in cohorts of up to 20 animals. A total of 4 mice were excluded due to health issues, and 5 were excluded due to failure to reach acyclicity. Final group sizes ranged from 10 to 13. During testing and analysis, experimenters were blinded to surgical and menopausal group.

### Menopause model

Beginning at ~ 2.5 months of age, mice were injected daily (i.p., daily for 20 days) with 4-vinylcyclohexene diepoxide (160 mg/kg) or vehicle (sesame oil) to induce ovarian follicular atresia. Cyclicity was assessed using vaginal cytology in which the vaginal canal was lavaged with 1xPBS and the cellular content was assessed. Acyclicity (post-menopausal status) was defined by 10 consecutive days of a mouse remaining in the diestrus stage of the estrous cycle. Mice that were given the 4-vinylcyclohexene diepoxide injections but did not go into menopause were excluded (a total of 5 mice out of 55 mice).

### VCID model

At approximately 6 months of age, mice received a unilateral common carotid artery occlusion (UCCAO; VCID group) surgery to elicit chronic cerebral hypoperfusion or sham surgery, as previously described [35–37]. Under isoflurane anesthesia, the right common carotid artery was ligated with two 6-0 silk sutures and cauterized (VCID group). The sham surgery consisted of exposing the carotid artery without ligation. Incision sites were closed with Vetbond, and the mice were given 100 µL of 0.03 mg/mL buprenorphine via subcutaneous injection twice per day for 3 days for analgesia.

### Glucose tolerance test

As previously described [37–39], mice were given a glucose tolerance test (GTT) to assess disturbances in glucose tolerance. The mice were fasted overnight, and their fasting blood glucose levels were measured (*t* = 0) using a glucometer (Verio IQ, OneTouch, Sunnyvale CA, USA) from their tail vein. Following an i.p. injection of 2 g/kg of glucose, blood glucose levels were measured at 15, 30, 60, 90, and 120 min post-injection to assess glucose tolerance.

### Behavior testing

Following a 1-week recovery post-GTT, mice were tested for exploratory activity and anxiety-like behavior in the open field, episodic-like memory in the novel object recognition test (NORT), spatial recognition memory in the object place test, spatial learning and memory in the Barnes maze and activities of daily living using a nest-building task. A minimum of 3 days of rest were allowed between each test (except for the open field/NORT/object place test since the open field also serves as acclimation for NORT). Videos were recorded of behavioral performance for open field, NORT, object place, and Barnes maze and analyzed using automated tracking software (ANY-maze 5.1, Stoelting, Wood Dale, IL, USA). For each test, mice were placed into the procedure room under dim light and allowed to acclimate for 1 h. Each test apparatus was cleaned with 70% ethanol between each mouse to remove olfactory cues.

#### Open field

The mice were placed in the test apparatus (box 495 × 495 mm) for 10 min. Distance traveled was used to determine the general activity levels of the animal. The percentage of time spent in the center of the arena was used to determine anxiety-like behavior.

#### NORT

NORT consisted of two, 5-min trials performed in the same open field arena. Each mouse was placed in a box with two identical rubber ducks (the familiar object) for 5 min. Time spent with each object was measured. Mice that did not explore both objects during the training session were excluded (a total of 5 mice). After 1 h, the mouse was again placed in the box for 5 min, but one of the ducks was replaced by a salt-shaker. Time spent with each object was again measured. Mice with intact episodic memory are likely to spend more time with the novel object (salt-shaker). Videos of the novel object recognition test were analyzed using a tracking software (ANY-maze 5.1, Stoelting, Wood Dale, IL, USA).

#### Object place test

The object place test consisted of two, 5-min trials performed in the same open field arena with visual cues (black tape) placed on the walls of the arena 24 h apart. Each mouse was placed in a box with two identical Rubik’s cubes for 5 min. Time spent with each object was measured. After 24 h, the mouse was again placed in the box for 5 min, but one of the objects was moved to a novel location. Time spent with each object was again measured. Mice with intact spatial recognition memory are likely to spend more time with the novel location object. Videos of the object place test were analyzed using a tracking software (ANY-maze 5.1, Stoelting, Wood Dale, IL, USA).

#### Barnes maze

Hippocampus-dependent spatial learning and memory were assessed using the Barnes maze. The Barnes maze was purchased through Anymaze (Catalog # 60170) and consists of a round arena with 20 holes around the periphery. This test took place over 5 days during which a mouse was trained to reach a target hole (1 out of 20 total holes in the table). All the holes have an insert except for the target hole which has an escape box attached to it. On the first day, the mice underwent a habituation trial during which the mouse was placed in a transparent beaker in the center of the table for 30 s. Then the beaker containing the mouse was slowly dragged to the target hole. The mouse was allowed up to 3 min to enter the escape box on its own before being placed in the escape box. The mouse was allowed to remain in the hole for 1 min. The mice were returned to their home cage for 30 min before the first training trial (Trial 1). Trial 2 and 3 took place on the second day, trial 4 and 5 took place on the third day, and the probe trial took place on the fifth day. During each trial the mouse was placed in a opaque 4″ diameter cylinder with a lid in the center of the table for 15 s before the mouse was released and allowed to explore the maze for up to 3 min or until the mouse entered the escape box. The mouse was allowed to remain in the escape box for 1 min undisturbed. During the probe trial, the escape box was replaced with an insert.

#### Nest building

Ability to perform activities of daily living was measured by a nest-building test at approximately 8.5 months of age. The nest building test was performed as previously described [40]. Mice were singly housed in Allentown cages with pine chip bedding and two pre-weighed Nestlets each. After 16 h (overnight), the mice were removed from their test cage and returned to group housing. Nests were rated on a 1–5 scale (with half-point scores allowed) based on published criteria [41] by 3 experimenters that were blinded to treatment group. The 3 ratings were averaged.

#### Blood flow analysis

Prior to euthanasia, blood flow was measured using laser speckle contrast imaging under isoflurane anesthesia. The skull was exposed, and mineral oil was applied. The dorsal surface of the brain was scanned. Blood flow was measured using a moorFLPI full field laser perfusion imager (Moor Instruments, Wilmington, DE, USA). The moorFLPI Review V4.0 software was used to compare blood flow in the right and left zone of anastomoses (ZOA) regions, which are adjacent to the temporal lobes. Since the UCCAO surgery was performed on the right side in VCID mice, blood flow is initially only reduced on the right side. The redundancy of the circle of Willis allows for some compensation, but overall cerebral blood flow is reduced particularly in the hemisphere ipsilateral to the occlusion. The percent differences were calculated using the following formula: [(right side blood flow intensity – left side blood flow intensity)/(left side blood flow intensity)] × 100.

### Tissue collection

Mice were anesthetized with isoflurane and euthanized by cardiac puncture at about 9.5 months of age. Mice were perfused with heparinized saline. Following euthanasia, the brain and reproductive organs were collected from each mouse. The weight of the uterus was measured. Brains from a subset of mice (*n* = 3–5/group) were placed in 4% paraformaldehyde for 24 h, then placed in 30% sucrose for 48 h. Brains from the remaining mice (*n* = 3–5/group) were dissected and regions were flash frozen. Once the brains sank to the bottom, they were frozen in OCT and stored at – 80 °C.

### Analysis of gene expression using qPCR

qPCR was performed according to our previously published methods [40]. Flash-frozen isolated brain regions (ipsilateral to VCID or sham surgery) were thawed and homogenized in 25–50 µL RNA-Later (45-R0901-100MLsigma). RNA was extracted from 25 µL of homogenate using the RNeasy^®^ Plus Mini Kit (Qiagen, Catalog number 74134). RNA concentrations were determined using ThermoScientific NanoDrop One and RNA was converted to cDNA using a High-Capacity cDNA Reverse Transcription Kit (Applied Biosystems, Catalog number: 4368814). The qPCR reactions were performed using TaqMan Gene Expression Master Mix (Applied Biosystems, Catalog number 4369016) in the presence of TaqMan Assays with primer/probes for ERα (Mm00433149_m1), ERβ (Mm00599821_m1), GPER1 (Mm02620446_s1), Cyp19a1 (Pn4351368), MBP (Mm01266402_m1), Olig2 (Mm01210556_m1), Iba1 (Mm00479862_g1), GFAP (Mm01253033_m1), and CD68 (Mm00839636_g1) as target genes. RPL13A (Mm05910660_g1) was used as the housekeeping gene. Data are represented as fold change using relative normalized expression compared to Control-Sham mice (ΔΔCq) using Bio-Rad CFX Maestro software.

### Statistical analysis

GraphPad Prism software (San Diego, CA, USA) was used for data analysis. Grubbs outlier tests were performed and detected outliers were excluded. To assess differences between groups in measures such as blood flow, uterine weights, and novel object recognition data, a two-way ANOVA [surgery (sham vs. VCID) × injections (oil vs. menopause)] with Tukey’s multiple comparisons was performed. For nest scores and LFB grades, which were both non-continuous variables, a Kruskal–Wallis test with Dunn’s multiple comparisons was used to assess differences between groups. Linear regression analyses were performed in order to assess whether any of the pathological results (e.g., blood flow deficit, uterine weight, LFB data) had an association with behavioral results.

## Results

### Animal models and timeline

To model menopause, mice were treated with 20 days of 4-vinylcyclohexene diepoxide injections to induce accelerated ovarian failure (acyclicity). To model VCID, mice underwent a unilateral common carotid artery occlusion surgery to induce chronic cerebral hypoperfusion. These mice were compared to vehicle treated and sham-operated controls. A study timeline is shown in Fig. 1A. To confirm the timing of menopause, we performed vaginal cytology and documented the amount of time it took the mice to reach acyclicity (between 60 and 105 days).

### Menopause contributes to metabolic impairments

**Fig. 2:**
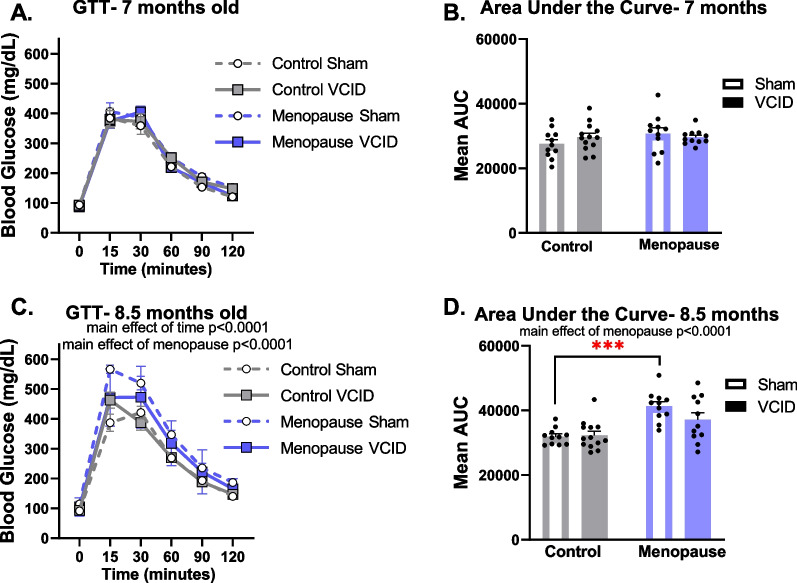
The accelerated ovarian failure model of menopause induced glucose intolerance over time. At 7 months old (4.5 months post first 4-VCD or vehicle injection), glucose intolerance was assessed with a GTT following a 16 h fast (**A**). Glucose clearance was gauged by concentrations of glucose in the blood measured over time (time 0 = fasting blood glucose). **B** Blood glucose concentration over time was used to calculate area under the curve (AUC). Glucose clearance (**C**) and intolerance (**D**, AUC) was also assessed at 8.5 months old (6 months post first 4-VCD or vehicle injection). Data are presented as mean + SEM, *p < 0.05, **p < 0.01, ***p < 0.001, ****p < 0.0001, 2-way ANOVA with Tukey’s multiple comparison test, (*n* = 10–13/group)

Menopause is associated with metabolic impairment in both humans [12, 42, 43] and rodents [18–20, 43–47]; therefore, we examined changes in weight, adiposity, and glucose tolerance. There was a main effect of menopause to increase body weight at the end of the study (*p* < 0.0001; Fig. 1B), weight gain over the course of the study (*p* < 0.001; Fig. 1C), as well as visceral fat pad weight (*p* < 0.01; Fig. 1D). When normalized to body weight, there was no main effect of menopause to increase visceral fat, however there was a main effect of VCID to lower normalized visceral fat levels (*p* < 0.01; Fig. 1E). There was a main effect of menopause to increase subcutaneous fat pad weight (Fig. 1F, G). A glucose tolerance test was performed to assess glucose intolerance at 4.5 months following the start of 4-vinylcyclohexene diepoxide injections (mice were 7 months old and roughly 1.5 months post-average onset of menopause), and there were no group differences (Fig. 2A, B). However, at 8.5 months of age there was a main effect of menopause to increase glucose intolerance (*p* < 0.0001; Fig. 2C, D). The area under the curve was calculated based on blood glucose over time and a main effect of menopause to increase glucose intolerance was found (*p* < 0.0001; Fig. 2D). Taken together, the data show that menopause led to metabolic impairments.

### The combination of VCID and menopause led to a greater array of cognitive deficits than VCID alone

**Fig. 3:**
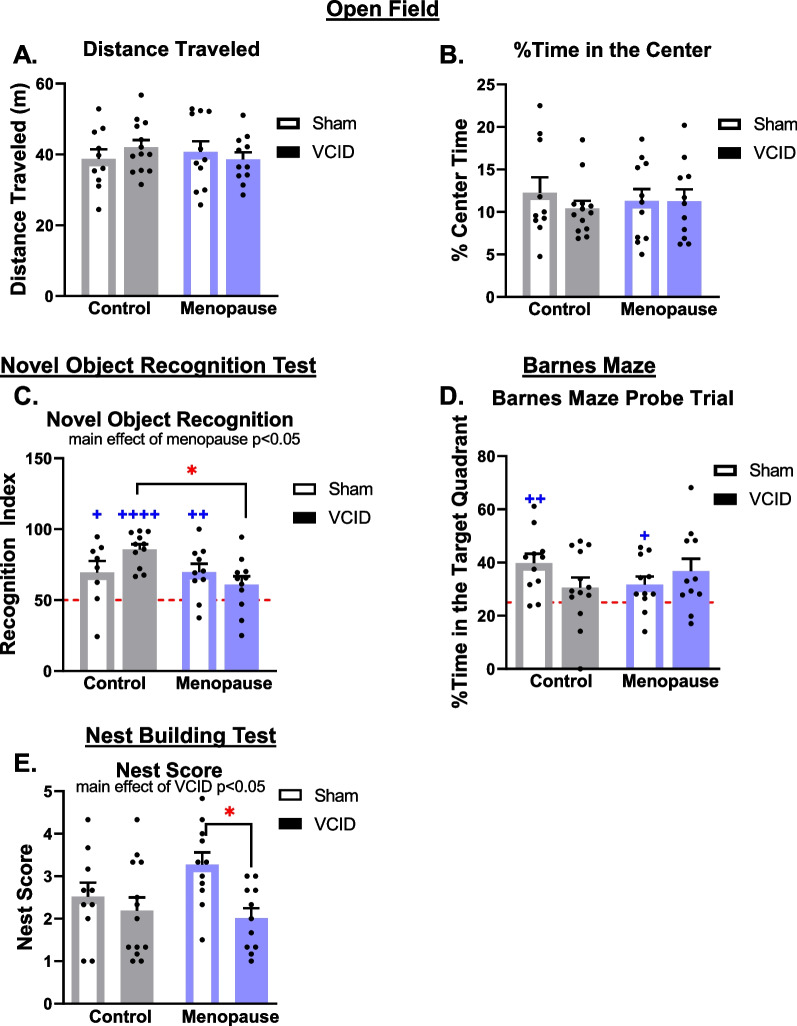
Post-menopausal VCID caused a wider array of cognitive deficits than VCID alone. General activity levels were gauged using the total distance traveled in the open field test (**A**). Anxiety-like behavior was measured as the % of the time that the animal spent in the center of the testing arena during the open field test (more time in the center = less anxiety-like behavior) (**B**). Episodic-like memory was assessed in the novel object recognition test (NORT) (**C**). Recognition index (% time spent with the novel object) was calculated. Performance not significantly greater than chance (red line = 50%) indicates impaired memory. Spatial memory was assessed in the probe trial of the Barnes maze (**D**), as % time spent in the target quadrant vs. chance. Performance not significantly greater than chance (red line = 25%) indicates impaired memory. The nest building task was used to assess activities of daily living (**E**). Nests were graded on 1–5 scale (average of scores by 3 experimenters blinded to treatment). Lower scores are indicative of impairment. Data are presented as mean + SEM, +*p* < 0.05, ++*p* < 0.01, +++*p* < 0.001, ++++*p* < 0.0001 t-test vs chance, and *p < 0.05, **p < 0.01, 2-way ANOVA with Tukey’s multiple comparison test (*n* = 8–13/group)

We have previously reported that VCID causes spatial and episodic-like memory impairments in middle-aged female mice [37]. It was unknown how menopause would impact cognitive impairments in VCID in young female mice. Here we show that in young females, neither menopause nor VCID altered baseline activity levels (distance traveled in the open field test, Fig. 3A) or anxiety-like behavior (% time spent in the center of the testing arena, Fig. 3B). The object place test was used to assess spatial recognition memory. No impairments or group differences were found in the recognition index (Additional file 1: Fig. S1A). However, when only examining post-menopausal mice, there was a significant negative relationship via linear regression analysis between the time in menopause and the recognition index (Additional file 1: Fig. S1B) indicating that mice that had spent more time in menopause at the time of the object place test had worse spatial recognition memory. Episodic-like memory was assessed using the novel object recognition test. Recognition index in the NORT (episodic-like memory; Fig. 3C), showed that all groups except for the post-menopausal VCID group demonstrated a preference for the novel object (Control-Sham, *p* = 0.0473; Vehicle-VCID, *p* < 0.0001; Menopause-Sham, *p* = 0.0077; Menopause-VCID, *p* = 0.0919). Group differences in episodic-like memory were additionally assessed using a 2-way ANOVA finding a main effect of menopause to decrease episodic-like memory (*p* = 0.0411). This effect of menopause was driven by poorer episodic memory in the Menopause-VCID group (interaction effect *p* < 0.05; post hoc test vs. Control-VCID group *p* < 0.05). The Barnes maze was used to examine spatial learning and memory. During spatial learning training trials, there was a main effect of trial on the time it took the mouse to reach the target hole (spatial learning, Additional file 1: Fig. S1C, *p* < 0.01), indicating that mice were able to learn the task. There were no effects of menopause or VCID on spatial learning. In the probe trial of the Barnes maze, only sham groups demonstrated a preference for the target hole quadrant (Fig. 3D, Control-Sham: *p* < 0.01, Menopause-Sham: *p* < 0.05), indicating that mice in the VCID groups (regardless of menopause) had impaired spatial memory. Activities of daily living were assessed using the nest building test. There was a main effect of VCID to impair nest building (Fig. 3E, p = 0.0107) and post hoc testing indicated that this was driven by impairment in the Menopause-VCID mice (*p* = 0.0235). Taken together, these results show that post-menopausal VCID leads to a wider array of cognitive impairments than VCID alone.

### Menopause causes mild changes in genes that are commonly associated with neuropathology

**Fig. 4:**
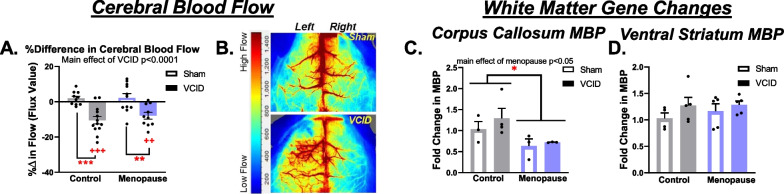
Neuropathology indicators associated with VCID and menopause. Cortical blood flow was measured using laser speckle contrast imaging at 9 months of age (3 months post-surgery) and a T-test performed against no difference in blood flow (**A**). The % difference in blood flow between the ischemic and non-ischemic hemispheres with a value closer to 0 indicating no difference in blood flow and a negative % difference indicating lower blood flow in the hemisphere ipsilateral to the occlusion (*n* = 11–13/group). Representative images are shown in **B**. White matter changes were also assessed by examining the expression of MBP, a marker of myelination. MBP expression normalized to RPL13a was measured in the corpus callosum (**C**) and in the ventral striatum (**D**). Data are presented as mean + SEM, +*p* < 0.05, ++++*p* < 0.0001 *T*-test vs chance and *p < 0.05, **p < 0.01, ***p < 0.001, 2-way ANOVA with Tukey’s multiple comparison test (*n* = 3–5/group)

We have previously found that in male mice, the UCCAO model of VCID leads to chronic cerebral hypoperfusion, increased Iba-1 and GFAP expression in the ischemic corpus callosum and increased Iba-1 expression in the hippocampus demonstrating increases in reactive changes [35]. Here we asked if menopause would exacerbate transcriptional indicators of pathological aspects of VCID including cerebral hypoperfusion, astrogliosis, and white matter changes. We found that the UCCAO model of VCID led to chronic cerebral hypoperfusion, with VCID groups having significantly lower blood flow in the right (ischemic) hemisphere at the end of the study (Control-VCID: *p* < 0.0001, Menopause-VCID: *p* < 0.05 via one-sample t-test vs. 0% change; main effect of VCID via 2-way ANOVA; *p* < 0.05; Fig. 4A). Representative laser speckle blood flow images are shown in Fig. 4B. We examined indicators of neuropathology in multiple brain regions (diagram in Additional file 1: Fig. S2A), including white matter (ventral striatum and the corpus callosum), the cortex (a brain region important for episodic-like memory), and the hippocampus (a brain region important for memory, especially spatial learning and memory). Glial fibrillary acidic protein (GFAP) is a marker of astrocytes, with heightened expression often being used as an indicator of astrogliosis. In sham operated mice, there was no effect of menopause on GFAP expression in the hippocampus (Additional file 1: Fig. S2B), cortex (Additional file 1: Fig. S2C), or ventral striatum (Additional file 1: Fig. S2D). There was a main effect of VCID to increase GFAP expression in the cortex (*p* < 0.01) and post hoc testing revealed that this was driven by increases in the Menopause-VCID group (*p* < 0.01). We additionally examined the expression of microglial marker Iba-1 and lysosomal phagocytosis marker CD68 in the cortex and found no significant effects (Additional file 1: Fig. S2E, F). White matter damage was assessed by grading the corpus callosum (visualized using Luxol fast blue) for damage (a score of 0 indicating no damage and 3 indicating the most damage). The regions were scored by 3 blinded observers, finding no main effect of menopause or VCID (Additional file 1: Fig. S3A, B). A linear regression showcasing the relationship between Luxol fast blue score (a measure of white matter damage) and NOR RI (a measure of episodic-like memory) is shown in Additional file 1: Fig. S3C, in which we observed a trend (*p* = 0.058) for more white matter damage being associated with worse episodic-like memory in post-menopausal mice but not in control mice. We additionally assessed the expression of white matter-related genes in the corpus callosum and the ventral striatum. The two white matter genes that we examined were myelin basic protein (MBP) and Oligodendrocyte transcription factor 2 (Olig2). MBP is a protein expressed by oligodendrocytes and is important to the formation of the myelin sheath. Decreased expression may indicate a deficit in myelination. Olig2 is a transcription factor expressed in both mature and precursor oligodendrocytes. Decreased expression may suggest decreased oligodendrocyte numbers. We found that there was a main effect of menopause to decrease MBP expression in the corpus callosum (Fig. 4C, p < 0.05). There were no group differences in MBP expression in the ventral striatum (Fig. 4D) or in Olig2 expression in the corpus callosum (Additional file 1: Fig. S3D). There was a main effect of VCID to increase Olig2 expression in the ventral striatum (Additional file 1: Fig. S3E, *p* < 0.05). To determine the presence of cerebral microbleeds, we used Prussian Blue histological staining, finding very few microbleeds in the ischemic (right) hemisphere (Additional file 1: Fig. S3F), with no differences between groups. Taken together, these results show that menopause caused only mild changes in transcriptional indicators of reduced myelination (in both sham and VCID mice) and increased astrogliosis (in VCID mice only) in select brain areas, with no changes in resting cerebral blood flow or overt white matter damage.

### Estrogen receptor expression persists after menopause in both the hippocampus and cortex

**Fig. 5:**
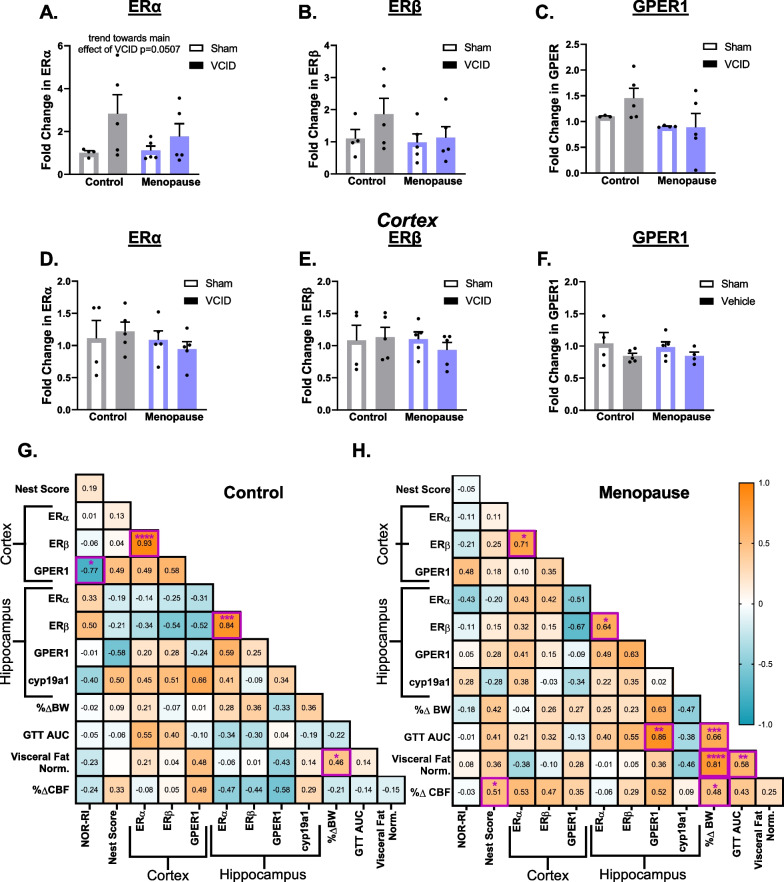
Estrogen receptor expression in the hippocampus and the cortex persists following menopause. The expression of estrogen receptor alpha (ERα) was assessed in the hippocampus (**A**) and the cortex (**D**). The expression of estrogen receptor beta (ERβ) was assessed in the hippocampus (**B**) and the cortex (**E**). The expression of G-coupled protein estrogen receptor (GPER1) was assessed in the hippocampus (**C**) and the cortex (**F**). Data are presented as mean + SEM, (*n* = 3–5/group). Using a correlation matrix, we compared relationships between cognitive, metabolic, and pathological factors including the expression of estrogen receptors for control (**G**) and menopause (**H**) groups separately. NOR-RI: novel object recognition test recognition index, a measurement of episodic-like memory; Nest Score: a measurement of activities of daily living; Cortex ERα: expression of ERα in the right (ischemic for VCID groups) cortex; Cortex ERβ: expression of ERβ in the right (ischemic for VCID groups) cortex; Cortex GPER1:expression of GPER1 in the right (ischemic for VCID groups) cortex; Hippocampus ERα: expression of Erα in the right (ischemic for VCID groups) hippocampus; Hippocampus Erβ: expression of ERβ in the right (ischemic for VCID groups) hippocampus; Hippocampus GPER1: expression of GPER1 in the right (ischemic for VCID groups) Hippocampus; Hippocampus cyp19a1: expression of aromatase in the right (ischemic for VCID groups) hippocampus; %ΔBW: % change in body weight from the beginning of the study; GTT AUC: area under the curve from the glucose tolerance test, high AUC indicates greater glucose intolerance; Visceral Fat Norm.: Visceral fat pad weight normalized to body weight; %ΔCBF: % difference in blood flow between the left and right ZOA; (*n* = 8–10/group for gene expression and *n* = 19–24 for behavior, metabolic, and blood flow measures). *p < 0.05, **p < 0.01, ***p < 0.001, ****p < 0.0001, significant correlation; Pearson *r* values are presented. Orange: positive correlation, Blue: negative correlation. Significant correlations are outlined in purple

Whether or not menopause effects in this model of VCID are associated with downregulation of estrogen receptor expression is unknown. Here we ask how menopause and VCID effect estrogen receptor expression in the hippocampus and the cortex and if estrogen receptor expression is correlated to cognitive and metabolic outcomes. We found a trend towards a main effect of VCID to increase estrogen receptor alpha (ERα) expression in the hippocampus (Fig. 5A, *p* = 0.0507). There were no group differences in estrogen receptor beta (ERβ) or G-protein coupled estrogen receptor 1 (GPER1) expression in the hippocampus (Fig. 5B–C). We additionally examined the expression of aromatase, the enzyme responsible for converting testosterone to estradiol, in the hippocampus and found no group differences (Additional file 1: Fig. S3G). There were also no group differences in cortical expression of ERα (Fig. 5D), ERβ (Fig. 5E), or GPER1 (Fig. 5F). These results indicate menopause and VCID did not downregulate estrogen receptors. We performed correlation analyses in control mice and menopause mice separately (Fig. 5G, H). In both groups, expression of ERβ was positively correlated with ERα in both the cortex (control group: *p* < 0.0001, menopause group *p* < 0.05) and the hippocampus (control group: *p* < 0.001, menopause group *p* < 0.05). Cortical GPER1 expression was negatively correlated with episodic-like memory (NOR RI) in control mice but not in menopause mice (*p* < 0.05), indicating that in pre-menopausal mice, higher cortical GPER1 expression was associated with worse episodic-like memory. Only in menopause mice, hippocampal GPER1 expression was positively correlated with glucose intolerance (GTT AUC; *p* < 0.01), indicating higher hippocampal GPER1 expression was associated with more severe glucose intolerance. In addition to these correlations between estrogen receptor expression and other measures, we also found that only in menopause mice, cerebral blood flow (%Δ ZOA) was positively correlated with activities of daily living (Nest Score, *p* < 0.05) and the degree of weight gain (%Δ BW, *p* < 0.05). Taken together, we found that estrogen receptors are still expressed in the brain regardless of menopause or VCID. However, menopausal mice had more correlations between measures of metabolic impairment and estrogen receptor expression (hippocampal GPER1 expression with GTT AUC) than mice that non-menopausal controls.

**Fig. 6:**
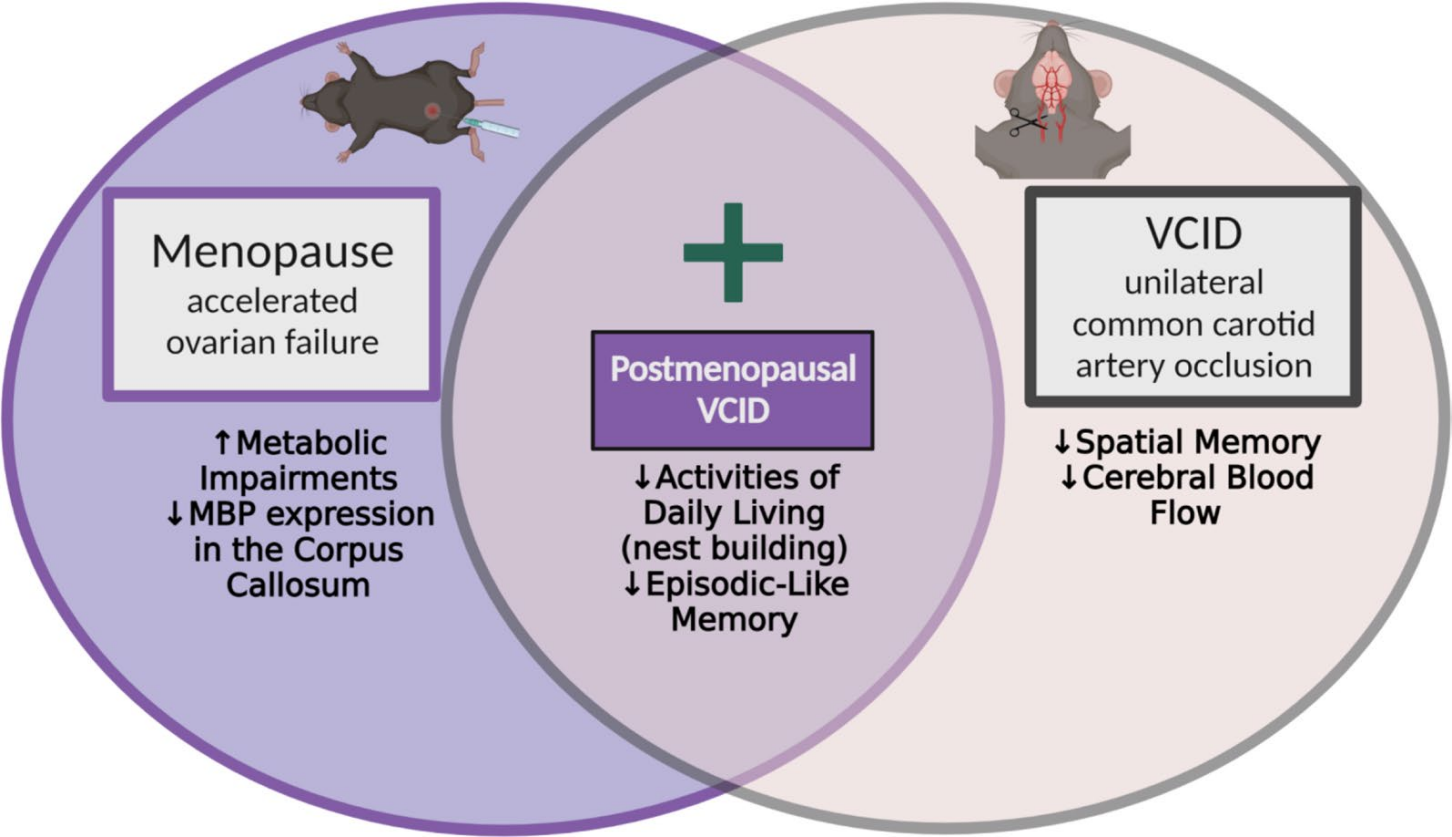
Summary of major findings. *MBP* myelin basic protein, a marker of myelination; Made using biorender.com

A summary of major findings is presented in Fig. 6.

## Discussion

Menopause is a significant event in women’s health and marks the end of menstruation. Because menopause usually occurs around the age of 55, the vast majority of women who suffer dementia are also post-menopausal. Unfortunately, endocrine aging is infrequently integrated into basic dementia research. In humans, systemic estrogen is much lower post-menopause. Estrogen has many significant, and protective effects on cerebrovascular function which we have previously reviewed [16]. There is evidence that estrogen protects cognitive function in rodent models of VCID [48] and human studies have pointed towards estrogen protecting the brain especially in the context of VCID risk factors [2, 13, 49], but it is unclear exactly how menopause contributes to cognitive aging. Here we aimed to address this gap in knowledge by examining the impact of menopause on cognitive impairment and pathology in a mouse model of VCID. We found that menopause produced various metabolic impairments, while VCID led to deficits in spatial memory. Furthermore, post-menopausal VCID mice have additional impairments in episodic-like memory and activities of daily living that were not observed in non-menopausal controls. We observed further potentially pathological effects of menopause and VCID, including alterations to white matter and astrocyte gene expression. Finally, we found that estrogen receptors are expressed at normal levels in the post-menopausal mouse brain. Taken together, post-menopausal VCID mice had a greater array of cognitive deficits than either menopause or VCID alone demonstrating additive negative outcomes.

In humans, menopause leads to metabolic changes including weight gain and visceral fat accumulation [42, 50]. Metabolic disease, a dementia risk factor itself, may be a greater VCID risk for women [3] and we have previously found that female mice are more sensitive to the negative cognitive consequences of a high fat diet [37, 40]. Understanding how menopause triggers metabolic changes and how menopause-induced metabolic impairments contribute to cognitive decline may unveil opportunities for prevention or further research. Here, we found that menopause mice gained more weight and had greater subcutaneous and visceral fat accumulation, as well as worse glucose tolerance. Ovariectomy (OVX) models of menopause have also repeatedly found weight gain [43, 44, 46, 47, 51–55]. Studies utilizing the 4-vinylcyclohexene diepoxide (4-VCD) menopause model have found similar effects [56, 57]. There is evidence supporting that the loss of systemic estrogen is at least partially responsible for post-menopausal weight gain: several studies have found that estrogen replacement alleviates menopause-induced weight gain [20, 53, 58, 59]. In addition to changes in body weight, menopause alters adipose distribution in humans, which may be of pathological relevance [42, 50]. Here we have found that menopause increased both visceral and subcutaneous fat mass. Menopause may contribute to weight gain through multiple mechanisms such as through estrogen’s actions on the liver [43], harmful changes to the microbiome [60], and alterations to leptin, a satiety hormone, entry into the brain [61]. Adipose tissue may also pose a greater health risk post-menopause. It was found that adipose tissue post-menopause or post-OVX was more inflammatory than that of controls [45–47]. While we are limited in our interpretation because we did not measure individual food intake, others have found that OVX leads to weight gain even with similar food intake [46]. Furthermore, we have also found no effect of menopause on baseline activity measured in the open field. Basic research into the effects of menopause, including our work, supports that weight gain following menopause may be more likely (and potentially more of a health risk)[45–47]. Disentangling the impact of post-menopausal weight gain from the direct effects of menopause on cognitive impairment is imperative; further research is needed to understand the effect of menopause on the brain.

Glucose is cleared from the blood stream as cells take it up for energy. Dysfunction in this process (such as glucose intolerance) is seen in prediabetes and diabetes—dementia risk factors that may be worse for women [3, 40]. Here, we found that menopause eventually but not immediately impaired glucose tolerance. Deficiencies in glucose tolerance appeared roughly 6 months following the first 4-VCD injection. Our findings are in line with those of Romero-Aleshire et al. who found that glucose intolerance was increased by menopause 26 weeks (6.5 months) after the first 4-VCD injection but not at earlier time points [19]. Here we found that in post-menopausal mice only, a wider array of metabolic impairments were positively correlated with each other (glucose intolerance was correlated with visceral adiposity and weight gain in post-menopausal mice). This demonstrates that post-menopausal mice with impairments in glucose intolerance were also more likely to be metabolically impaired in other measurements. Studies investigating the connection between menopause and impaired glucose tolerance point toward the involvement of estrogen. Replacing estrogen in the accelerated ovarian failure model improves glucose tolerance [19]. Estrogen acts on many aspects of metabolism and energy-control at the cell level: regulating the function of insulin production by pancreatic beta-cells and insulin sensitivity [45]. These data suggest that metabolic dysfunction, including weight gain, increased visceral and subcutaneous adiposity, and impaired glucose tolerance may occur following menopause.

We have previously shown that VCID, modeled by the common carotid artery occlusion surgery, leads to deficits in episodic-like memory in middle-aged female but not male mice [37]. Here we ask if VCID in female mice is made worse by menopause. We found that cognitive impairment was affected by VCID and menopause. VCID alone led to impairments in spatial memory (Barnes maze) while menopause in VCID mice led to additional impairments in episodic-like memory (NOR) and activities of daily living (nest building). Others have examined cognitive effects of the accelerated ovarian failure model of menopause at baseline and have found no deficits [34]. While others have examined the cognitive effects of an OVX model of menopause, we are the first to examine the cognitive impact of this more clinically relevant menopause model on cognitive impairment in VCID. However, in a study comparing cognitive effects of the OVX and accelerated ovarian failure models in rats, the accelerated ovarian failure model trended towards greater memory deficits [62]. Cognitive deficits due to OVX are well established [55, 63–66]. There is evidence that cognitive effects of OVX are mediated, at least in part, by changes to systemic estrogen levels as studies have found that estrogen replacement following OVX ameliorates cognitive deficits [48, 64]. Our findings support that menopause and VCID both contribute to cognitive deficits. It is yet to be determined if these effects can be reversed by estrogen in the context of VCID.

Standard aspects of VCID pathology include deficits in cerebral blood flow, white matter damage, and astrogliosis. In this work, we asked if menopause exacerbates these pathological impairments and if these effects vary by brain region. Unsurprisingly, VCID produced deficits in resting cerebral blood flow. However, contrary to our hypothesis, these deficits were not exacerbated by menopause. While we did not observe effects of menopause on resting cerebral blood flow, we did find that mice that had smaller reductions in cerebral blood flow were more likely to have higher scores in nest building. This suggests that preserving blood flow may protect against impairment in the ability to perform activities of daily living. No prior studies have examined chronic cerebral hypoperfusion in this model of menopause. While we did not observe an effect of menopause on blood flow in the cortical surface, hypoperfusion may still be exacerbated in other brain regions. We have previously shown, using MRI, that the UCCAO model of VCID leads to hypoperfusion in deeper brain areas such as the hippocampus [35]. Recently, Blackwell et al. showed that the accelerated ovarian failure model of menopause impairs cerebrovascular function in cerebral parenchymal arterioles isolated from deeper brain structures (below the corpus callosum) [34]. Interestingly, they too found that resting cerebral blood flow was unaltered, but rather it was the evoked responses to whisker stimulation that were impaired [67]. Further studies are needed to determine if menopause may alter blood flow in deeper structures or in response to evoked stimulus following UCCAO.

White matter damage is a hallmark of VCID as it is particularly susceptible to damage from hypoperfusion. Here, we asked if and how prolonged hypoperfusion impacts markers of myelination in key white matter regions and if menopause increases damage to these areas. Surprisingly, we saw an effect of menopause rather than of VCID to alter white matter gene expression. We found that menopause decreased MBP (myelin basic protein) expression in the corpus callosum. MBP is a protein that is involved in adhering the layers of myelin together and a decrease in expression could indicate less myelination. Deficits in MBP expression could indicate white matter damage but increases could also represent compensation and/or remyelination. While we did not find robust white matter changes at 3 months following the surgery (assessed via Luxol fast blue), it is possible that there were earlier changes in MBP expression due to VCID that resolved prior to our 3 month timepoint. While ours is the first study to examine changes to the expression levels of white matter genes in the accelerated ovarian failure model of menopause, we are limited in our interpretation as we are examining a snapshot in a dynamic process of gene expression 3 months following the onset of hypoperfusion (the VCID surgery). The effects of chronic cerebral hypoperfusion on white matter have been thoroughly studied in male rodents. For example, UCCAO decreased white matter fiber density [68] and MBP protein expression 6 weeks post-surgery in the corpus callosum of male mice [69]. However, female mice were not included in that study. Further highlighting changes in white matter over time, middle cerebral artery occlusion in male rats caused white matter damage evident by decreased myelin density in the corpus callosum and the striatum at 1 month post-occlusion, but not at 2 months post-occlusion, suggesting that there is white matter repair following hypoperfusion [70]. Additionally, estrogen was found to have a protective effect against hypoperfusion-induced white matter damage in male rats [48]. Human data suggest that white matter is at greater risk following menopause. Post-menopausal women have more white matter hyperintensities than either men or pre-menopausal women [71]. This may begin during peri-menopause, as more hot flashes are associated with more white matter hyperintensities [14]. Further, work from the Brinton lab has shown that during the menopausal transition the female brain undergoes a metabolic shift where ketones bodies (extracted form white matter) are preferentially used as a fuel source, leading to white matter loss [72, 73]. Menopause may affect white matter through the effects of estrogen, as OVX models of menopause have shown that estrogen replacement improves white matter sheath volume [74–76]. Our work indicates that there are small white matter changes following menopause in the corpus callosum which may impact cognitive function. Further studies examining the dynamics of post-menopausal white matter change would greatly benefit the field.

Astrogliosis is an astrocytic damage-response mechanism which can sometimes potentiate damage and contribute to a pathological state. Here we examined a surrogate indicator of astrogliosis through the expression of GFAP in the hippocampus, cortex, and ventral striatum. We found no differences in the hippocampus. In the cortex, VCID increased GFAP expression (which could be indicative of increased astrogliosis). This difference was driven by the post-menopausal VCID group, suggesting that menopause may exacerbate cortical astrogliosis in VCID mice. Cortical brain regions are critical for episodic-like memory, which we found deficient in the post-menopausal VCID group. Increased astrogliosis may be involved in differences in cognitive deficits in these mice, but more in-depth studies would be needed to explore this. We have previously shown that chronic cerebral hypoperfusion increases astrogliosis in the corpus callosum [35]. Others have found that chronic cerebral hypoperfusion (modeled with a MCCAO surgery) increases astrogliosis in the cortex and striatum of male rats [77]. We are the first to examine GFAP expression following the accelerated ovarian failure model of menopause. These data indicate that menopause increased GFAP in the cortex of mice with VCID, highlighting a potential pathological difference that may hint at mechanisms involved in cognitive differences to explore in future studies.

Several mechanisms through which menopause may exacerbate chronic hypoperfusion-induced cognitive deficits or contribute to cognitive dysfunction were preliminarily explored in the current study, including reductions in cerebral blood flow, white matter damage, and expression of genes related to astrogliosis and microgliosis. Menopause had modest effects on white matter and astrocyte-related gene expression in some regions (corpus callosum and cortex, respectively), but not others. Further, no alterations in the microglia/macrophage marker IBA1 were found. Thus, further studies are needed to identify mechanisms. Other potential mechanisms such as cerebrovascular inflammation [78–81], altered cerebrovascular or blood brain barrier function [34], oxidative stress [63–65], and cell survival [82] could contribute to cognitive deficits observed in post-menopausal VCID and would be interesting to explore in future studies. Finally, as mentioned above in relation to white matter changes, all neuropathology assessments were performed at a single timepoint, and thus important acute or more long-term changes could have been missed.

Estrogen’s protective actions in the brain work via the molecule’s innate antioxidant properties, but also through rapid and genomic effects mediated by its receptors: ERα, ERβ, and GPER1. In rodent models, both OVX and systemic estrogen treatment have been shown to alter hippocampal gene expression (23) and ERα receptors in the brain [24] and in cerebral microvessels [25]. Sustained expression of estrogen receptors in the brain and the ratio of the different types of estrogen receptors would additionally impact the effectiveness of estrogen replacement therapies. While ERβ has been reported to be unchanged in the accelerated ovarian failure menopause model [83], effects on ERα and GPER1 were unknown and the effects in the context of VCID were unknown. We found no significant group differences in estrogen receptor expression in the hippocampus and in the cortex, demonstrating the ERα, ERβ, and GPER1 are all expressed in the hippocampus and cortex following menopause and UCCAO at normal levels. Others have examined the impact of OVX on brain estrogen receptor expression, finding that OVX in rats leads only to a decrease in ERα in the hippocampus but not ERβ [84]. This is in line with the hypothesis that prolonged estrogen deprivation can change the ratio of ERα to ERβ and thus reduce the beneficial effects of estrogen on the brain. Prolonged estrogen deprivation has been shown to have different effects on gene expression than early estrogen deprivation [18], thus in the current study where we only assessed long-term changes, we cannot rule out any short-term changes in estrogen receptors that may have occurred. While we examined changes to the expression of ERalpha, ERbeta, and GPER1, an additional unsequenced estrogen receptor (Gq-mER) could also be involved in protecting hippocampal function in VCID. Delivery of STX, a selective estrogen receptor modulator that binds to Gq-mER and not to classical estrogen receptors, to the brain increased hippocampal neuronal cell survival in rodent experimental ischemia [85]. Taken together, our findings indicate that in the hippocampus and cortex, estrogen receptor expression is conserved long-term following menopause.

In summary, using mouse model of menopause (accelerated ovarian failure) and VCID, we found that post-menopausal VCID females showed a wider array of cognitive deficits, compared to those with menopause or VCID alone. VCID specifically impaired spatial memory (Barnes Maze) in both control (pre-menopausal) and post-menopausal groups. Post-menopausal VCID impaired both episodic-like memory (NOR) and activities of daily living (nest building), which highlights that menopause exacerbated VCID-related cognitive deficits. Menopause did not alter cerebral blood flow, but caused modest changes in some white matter and astrocyte related genes in brain region-specific manner. In line with previous studies, we found that menopause consistently led to increased metabolic dysfunction including weight gain, visceral and subcutaneous fat pad weights, and impairments in glucose tolerance. Finally, we found that estrogen receptor expression was maintained in the post-menopausal brain.

## Perspectives and significance

The vast majority of women suffering from VCID are post-menopausal, yet animal studies of VCID rarely take endocrine aging into account. Here, we demonstrate the importance of including endocrine aging/menopause in the study of dementia, as we show that menopause leads to a wider array of cognitive impairments in a mouse model of VCID. This work supports that menopause may be an accelerating factor when it comes to cognitive aging and VCID in particular. Future studies assessing how the effects of menopause and aging interact to affect dementia (including other dementia types such as Alzheimer’s and mixed dementias) are needed. Due to the significant metabolic effects of menopause, further studies are underway to investigate the added metabolic challenge of a high fat diet. Additionally, we did not attempt to rescue the cognitive and metabolic impairments with administration of estrogen, but this is an important next step in understanding the role of estrogen in post-menopausal VCID. Our finding that estrogen receptor expression is maintained following menopause in key brain areas associated with cognitive function may encourage further research into manipulating brain estradiol levels to ameliorate cognitive impairment.

## Supplementary Information

**Additional file 1.** Methods and Figures.

## Data Availability

The datasets used and/or analyzed during the current study are available from the corresponding author on reasonable request.

## Abbreviations

AUC: Area under the curve
CD68: Cluster of differentiation 68
ERα: Estrogen receptor α
ERβ: Estrogen receptor β
GFAP: Glial fibrillary acidic protein
GPER1: G-protein coupled estrogen receptor 1
GTT: Glucose tolerance test
IBA1: Ionized calcium-binding adaptor molecule 1
LFB: Luxol fast blue
MBP: Myelin basic protein
NOR: Novel object recognition
NORT: Novel object recognition test
Olig2: Oligodendrocyte transcription factor 2
OVX: Ovariectomy
RI: Recognition Index
RPL13A: Ribosomal protein L13a
SEM: Standard error of the mean
UCCAO: Unilateral common carotid artery occlusion surgery
4-VCD: 4-Vinylcyclohexene diepoxide
VCID: Vascular contributions to cognitive impairment and dementia
ZOA: Zone of anastomosis
