# Supplementary material for: Menopause causes metabolic and cognitive impairments in a chronic cerebral hypoperfusion model of vascular contributions to cognitive impairment and dementia
Source: Biol Sex Differ. 2023 May 23;14:34. doi: 10.1186/s13293-023-00518-7 (PMC10204285; doi:10.1186/s13293-023-00518-7)
Supplement: Supplementary file 1 — Additional file 1. Methods and Figures. [file 13293_2023_518_MOESM1_ESM.pdf]

Additional file 1 METHODS

Sectioning and Luxol Fast Blue (LFB) Staining: LFB is a lipid-binding histological stain used to visualize myelin. Brains from 21 mice were frozen in OCT and cut into 10 µm sections using a Leica CM1950 cryostat and were then immediately slide-mounted. LFB solution was made by diluting Solvent blue 38 (Sigma, Lot # SHBM2732) in ethanol and acetic acid. The slides incubated in LFB for 1 hour at 62 degrees Celsius. They were then washed in lithium carbonate (Sigma Aldrich Lot # 2WXBC9892V) to differentiate white matter from gray matter. The slides were then washed, dehydrated, and coated with xylene. Slides were sealed using Cytoseal XYL (Thermo Scientific, Lot # 535107). They were imaged using NDP Nanozoomer S60 Bright Field by the Albany Medical Center Pathology Core. Images were scored by 3 blinded lab members according to the following criteria. Sections with 0-10% discoloration in the corpus callosum received a Grade 0. Grade 1 corresponded to 10-33% damage to the corpus callosum. Grade 2 corresponded to 33-67% damage to the corpus callosum. Grade 3 corresponded to 67-100% damage to the corpus callosum.

Histological detection of cerebral microbleeds: Sections used for detection of microbleeds were mounted on slides and stained with Prussian Blue for 20 minutes and nuclear fast red for 5 minutes (Iron Stain, ab 150674, Abcam). Slides were cleared in xylene followed by cover slipping with mounting medium (Cytoseal XYL, 8312-4, Thermo Scientific). Slides were imaged in the Albany Medical Center Pathology Core with a NDP Nanozoomer S60 Bright Field. Total numbers of cerebral microbleeds and numbers in the ischemic and non-ischemic hemisphere, were counted in all groups by a blinded observer.

Additional file 1 FIGURES

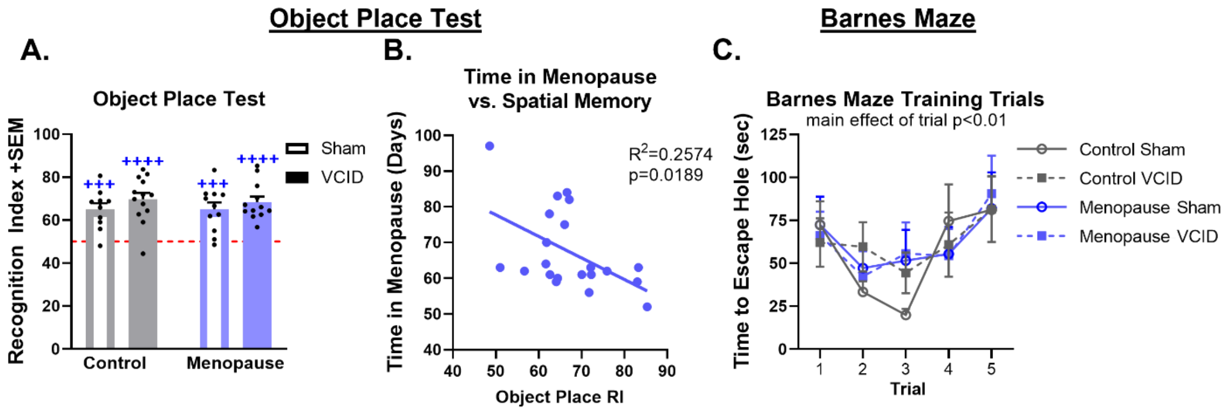

Figure S1. All mice had intact spatial recognition memory in the object place recognition test. Spatial memory was assessed in the object place recognition test. Recognition index (% time spent with the novel place object) was calculated (**A**). Performance not significantly greater than chance (50%, indicated by the red line) indicates impaired memory. The amount of time that mice were in menopause at the date of the object place test significantly correlated with object place recognition index (RI), (**B**) linear regression n=21). Spatial learning was assessed using the Barnes Maze. Five trials **(C)** assessed spatial learning via time to reach the escape hole (faster escape= better performance). A 3-way ANOVA was performed. Data are presented as mean + SEM, +++$p<0.001$ ++++$p<0.0001$ T-test vs chance, and *$p<0.05$, 2-way ANOVA with Tukey's multiple comparison test (n=8-13/group).

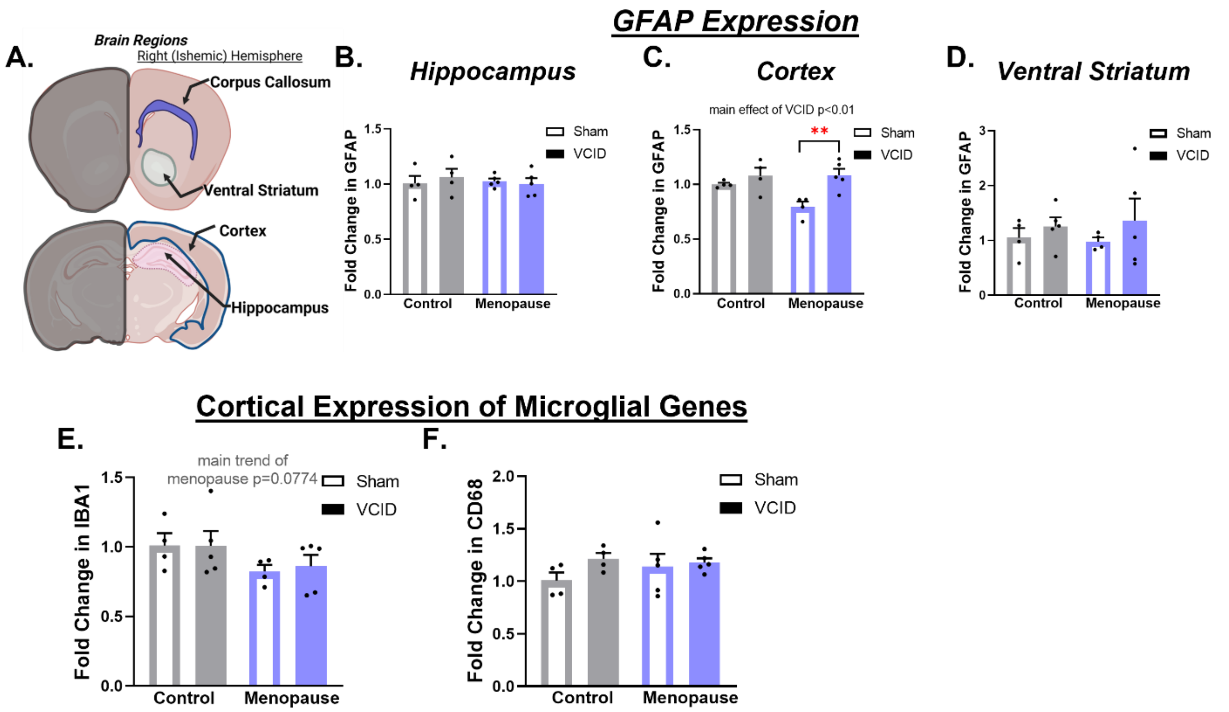

Figure S2. VCID increases GFAP expression in the brain. Diagram of brain regions isolated for gene expression analysis made using Biorender.com (**A**). GFAP expression normalized to RPL13A expression was measured in the hippocampus (**B**), cortex (**C**), and ventral striatum (**D**). Expression of microglia markers **(E)** Iba1 and **(F)** CD68 in the right (ischemic for VCID groups) cortex. All expression levels were normalized as to RPL13a and are shown as fold change from the sham vehicle group. Data are presented as mean + SEM, , **p<0.01 2-way ANOVA with Tukey's multiple comparison test (n=3-5/group).

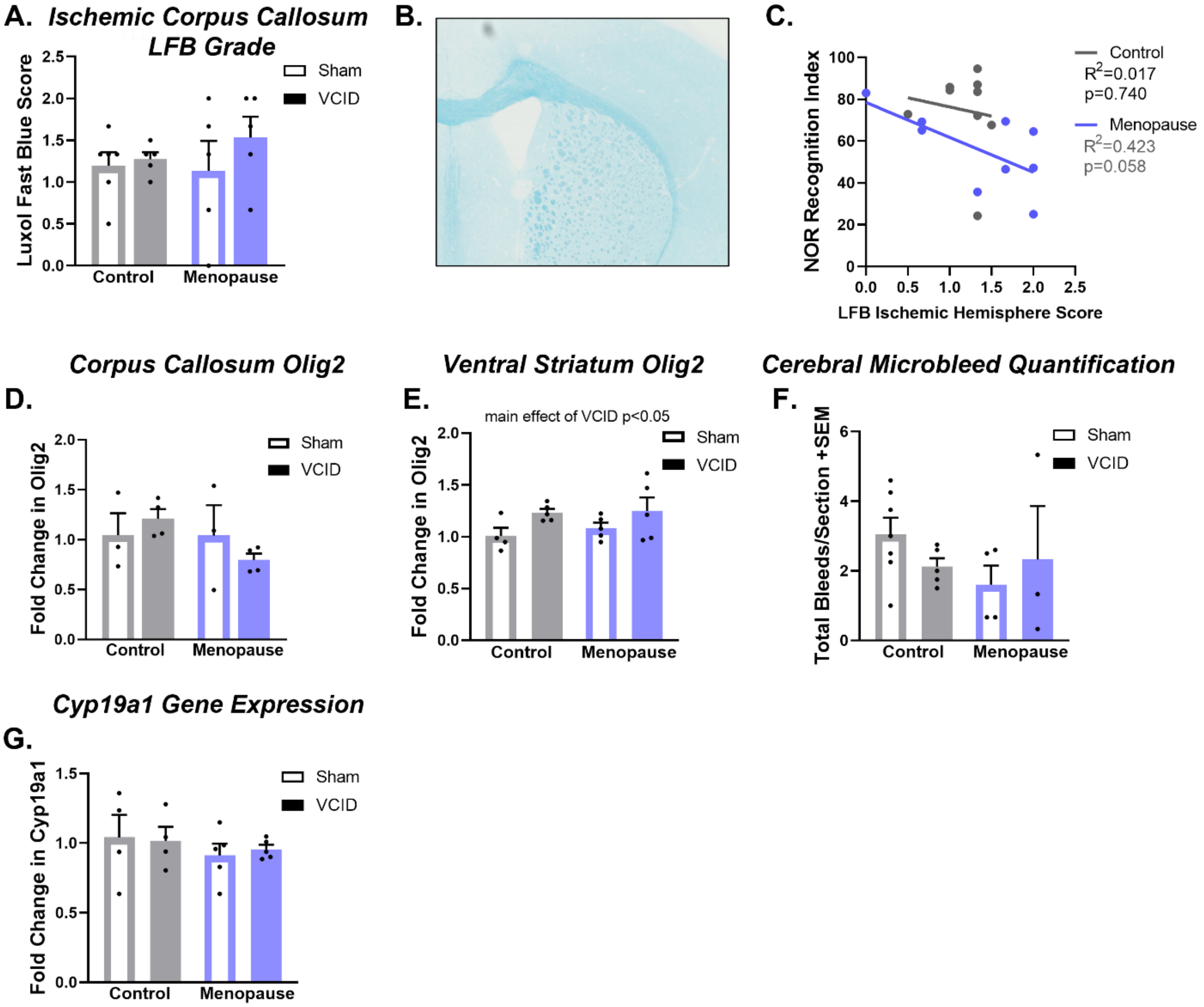

Figure S3. White matter changes associated with VCID and menopause. White matter damage in the corpus callosum was assessed using Luxol Fast Blue (LFB) histology and the degree of damage was graded by 3 blinded observers (a higher score=more damage) **(A)**. Representative image of LFB staining (**B**). Luxol fast score trended towards a significant relationship with the novel object test recognition index (episodic-like memory) in in menopause mice only **(C)**, linear regression analysis; n=8-9/group). Olig-2 expression normalized to RPL13a was measured in the corpus callosum **(D)** and in the ventral striatum **(E)**. Brain sections were stained with Prussian blue and cerebral microbleeds were hand counted by a blinded observer **(F,** n=3-6/group). The expression of aromatase (Cyp19a1) was assessed in the **(G)** hippocampus. Expression was normalized as to RPL13a and are shown as fold change from the sham vehicle group. Data are presented as mean + SEM, +p<0.05, ++++p<0.0001 T-test vs chance, *p<0.05, **p<0.01 2-way ANOVA with Tukey's multiple comparison test. Data are presented as mean + SEM, 2-way ANOVA with Tukey's multiple comparison test (n=3-5/group).
